# Time-restricted feeding promotes glucagon-like peptide-1 secretion and regulates appetite via tryptophan metabolism of gut *Lactobacillus* in pigs

**DOI:** 10.1080/19490976.2025.2467185

**Published:** 2025-02-14

**Authors:** Qiuke Li, Ding Tan, Shijie Xiong, Kaifan Yu, Yong Su, Weiyun Zhu

**Affiliations:** a Laboratory of Gastrointestinal Microbiology, Jiangsu Key Laboratory of Gastrointestinal Nutrition and Animal Health, College of Animal Science and Technology, Nanjing Agricultural University, Nanjing, China; b National Center for International Research on Animal Gut Nutrition, Nanjing Agricultural University, Nanjing, China

**Keywords:** Appetite, time-restricted feeding, GLP-1, intestinal stem cells, gut microbiota

## Abstract

Previous clinical trials have shown that time-restricted feeding can be involved in regulating the metabolic health of humans and animals. However, the underlying mechanism has not been fully explored. In this study, the pig model was employed to simulate four prevalent human eating habits, with the aim of investigating the impact of gut microbiota and microbial metabolites on gut hormone secretion and appetite regulation. Compared to the *ad libitum* feeding (ALF) pattern, three time-restricted feeding patterns reduced total food intake and eating time. Meanwhile, three time-restricted feeding patterns induced elevated levels of serum and hypothalamic glucagon-like peptide-1 (GLP-1), while suppressing reward-related circuits in the hypothalamus. It is noteworthy that the early time-restricted feeding (eTRF) pattern increased the number of intestinal enteroendocrine cells (EECs) compared to ALF. Metagenomic and metabonomic analyses revealed that three time-restricted feeding patterns induced colonization of *Lactobacillus* and significantly increased the levels of its metabolite, indole-3-lactic acid (ILA). Dietary supplementation with ILA exhibited an increasing trend in fasting serum GLP-1 level of piglets. *In vitro* studies with pig intestinal organoids showed the *Lactobacillus* metabolite ILA enhanced GLP-1 secretion through the promotion of intestinal stem cell differentiation into EECs, rather than activating the ability of EECs to secrete GLP-1. Overall, time-restricted feeding promoted GLP-1 secretion and affected long-term appetite regulation by promoting the colonization of *Lactobacillus* and modulating microbial tryptophan metabolism.

## GRAPHICAL ABSTRACT

**Figure:**
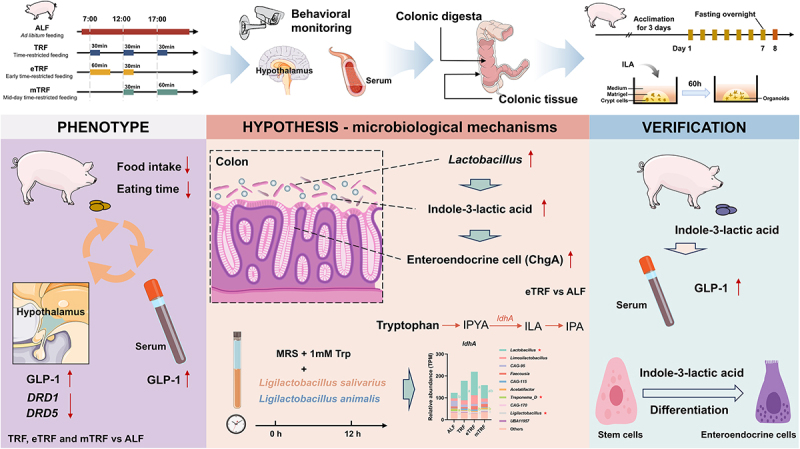

## Introduction

Reducing dietary energy intake to induce negative energy balance is one of the current recommendations for weight management.^1^ However, calorie-restricted weight loss leads to rebound weight gain in a ghrelin-dependent manner.^2^ Time-restricted feeding is a viable fasting regimen that confines daily food consumption to a 4- to 12-h window.^3^ It is more tolerable for humans over long periods than caloric restriction, which influences energy balance by regulating appetite and energy expenditure.^4^ Restricting food intake to an earlier time of the day (dinner before 15:00; “early time-restricted feeding” [eTRF]) reduces the desire and capacity to eat and increases the sensations of fullness in the evening.^5^ Compared to a large dinner, consuming a large breakfast has been shown to diminish appetite and feelings of hunger, which may be attributed to the reduction in 2-h postprandial ghrelin levels, increases in peptide YY (PYY) and GLP-1, as well as prolonged gastric emptying duration.^6^ However, restricting food intake to a late time of the day increases hunger sensations and the 24-h ratio of ghrelin and leptin, while decreasing lipolysis and increasing adipogenesis in adipose tissue.^7^ The feeding-fasting cycle, which coordinates with circadian rhythms of the body, transmits peripheral energy signaling through hormones and visceral signals to regulate food intake.^8^ However, the underlying mechanisms involved in the regulation of time-restricted feeding patterns on appetite remain inadequately elucidated.

The appetite control systems, encompassing homeostatic and reward-related circuits, are activated by hormones released from various tissues to transmit metabolic signaling related to energy storage and nutritional status.^9^ Gut is the first organ to perceive alterations in the timing of food intake and contains the largest population of hormone-producing cells in the body.^8^ Nutrient intake induces gut bacteria growth, leading to bacterial proteome changes that stimulate GLP-1 and PYY for activation of host satiety pathways.^10^ It is noteworthy that the GLP-1 signaling has been considered a promising approach for treating overeating disorders.^11^ The release of GLP-1 occurs in the distal intestine and undergoes rapid degradation mediated by dipeptidyl peptidase-4 (DPP-4). In addition to DPP-4 inhibitors and GLP-1 receptor agonists, augmenting endogenous GLP-1 secretion represents an efficacious strategy for elevating endogenous levels.^12^ The number and function of enteroendocrine cells (EECs) is closely related to the endogenous gut hormone secretion.^13^ The gut microbiota exerts a profound impact on the differentiation of intestinal stem cells.^14^ This interplay between the microbiota and the intestinal epithelium is critical for maintaining the physiological functions of the host.^15^ Gut microbiota-derived short-chain fatty acids (SCFAs) have been reported to suppress L cell function and GLP-1 secretion by promoting epithelial O-GlcNAcylation.^13^ Moreover, indole-3-acetic acid (IAA) enhances stem cell differentiation toward L cells and promotes GLP-1 secretion to prevent glucose metabolism disorders induced by total parenteral nutrition.^16^ However, the impact of time-restricted feeding on endogenous GLP-1 secretion through modulation of gut microbes has not been well explored.

Most previous studies on time-restricted feeding have utilized mouse models or human clinical trials. But it is important to note that the circadian rhythms of mice differ from those of humans. In contrast, the circadian rhythms, omnivorous fasting behavior, digestive tract physiological structure, and microbial functional pathway catalog of pigs closely resemble those of humans.^17,18^ Consequently, pigs are regarded as an ideal animal model for addressing the challenges associated with human live sample collection and the constraints of experimental design.^19,20^ Here, growing pigs were utilized to conduct the time-restricted feeding experiments. It is worth noting that the most popular time-restricted feeding patterns for the general public are 4-h time-restricted feeding pattern (the warrior diet, 20-h fasting duration) and 6-h time-restricted feeding pattern (the 18:6 diet, 18-h fasting duration).^20^ In this study, four feeding patterns were implemented, closely resembling typical human fasting durations. These included the *ad libitum* feeding (ALF) pattern to mimic irregular eating habits, the time-restricted feeding (TRF) pattern to mimic the conventional three-meal pattern (13.5-h fasting duration), the early time-restricted feeding (eTRF) pattern to mimic the pattern of skipping dinner (18.5-h fasting duration), and the mid-day time-restricted feeding (mTRF) pattern to mimic the pattern of skipping breakfast (18-h fasting duration). And the multi-omics analysis technology was used to investigate the potential microbiological mechanism underlying gut hormone secretion and appetite regulation through time-restricted feeding. These findings provide a novel perspective on the role of gut-brain communication in the regulation of appetite.

## Material and methods

### Time-restricted feeding experimental design and sample collection

**Figure 1.**
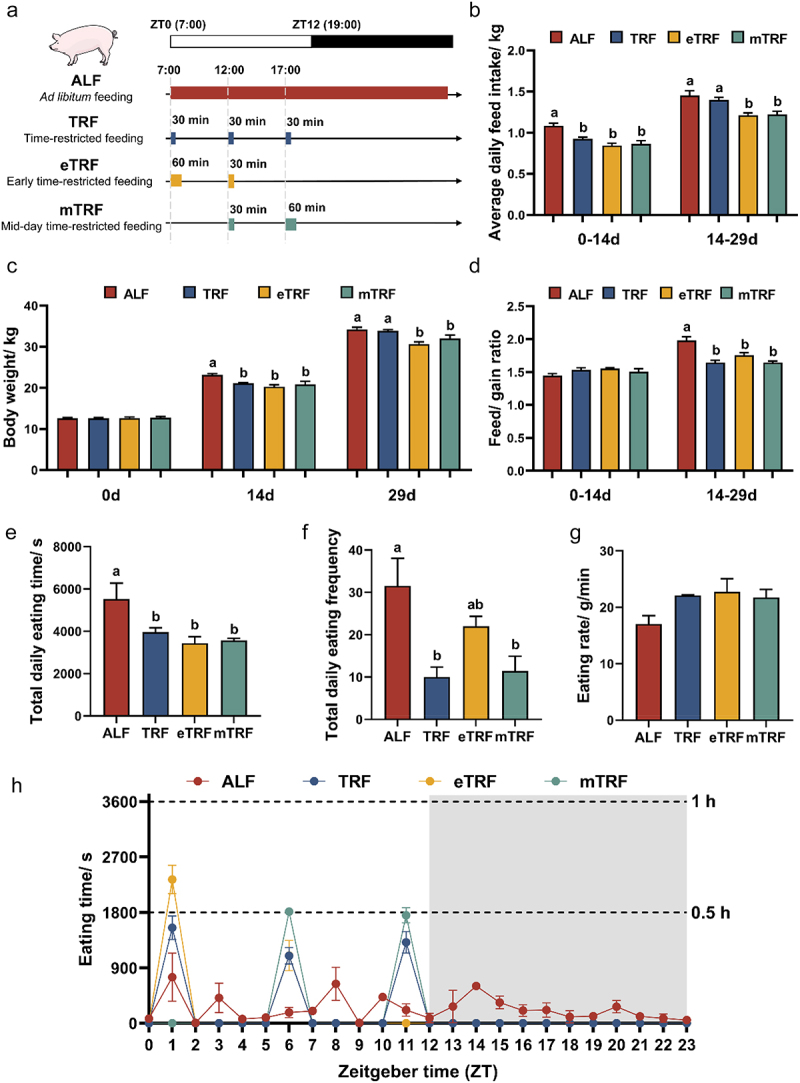
Effects of feeding patterns on growth performance and eating behaviors. (a) Experimental procedure. (b) Average daily feed intake (*n* = 7). (c) Body weight (*n* = 7). (d) The ratio of feed and gain (*n* = 7). (e) Total daily eating time (*n* = 3). (f) Total daily eating frequency (*n* = 3). (g) Eating rate (*n* = 3). (h) The distribution of eating time throughout the day (*n* = 3). The gray shaded area represents the dark-phase periods. Data are presented as the mean ± SEM. Different letters denote statistically significant differences among the groups (*p* < 0.05).

The animal procedure was carried out according to the guidance of the Animal Care and Use Committee of Nanjing Agricultural University (SYXK2019–0066) and implemented based on the standard of Experimental Animal Care and Use Guidelines of China (EACUGC2018–01). To investigate the potential associations between time-restricted feeding and appetite regulation, 28 healthy male Duroc × Landrace × Large White pigs (12.66 ± 0.11 kg) were randomly assigned to four groups, including ALF, TRF, eTRF and mTRF groups. Each group consisted of 7 replicates (pens) with 1 pig per pen. All pigs were subjected to a 12-h light/12-h dark cycle lighting protocol (Figure 1a), with the light period commencing at Zeitgeber time 0 (ZT0, 7:00) and ending at ZT12 (19:00). In the ALF group, pigs were allowed sufficient time for eating without any restrictions. The TRF group had a restricted eating window of 30 min at ZT0 (7:00), ZT5 (12:00), and ZT10 (17:00). The eTRF group had an eating window of 60 min at ZT0 (7:00) and 30 min at ZT5 (12:00). The mTRF group had a half-hour eating window at ZT5 (12:00) and a one-hour window at ZT10 (17:00). The total feeding duration for the three time-restricted feedings was 90 min per day. This duration was based on our previous studies, which found that the total eating time of pigs did not exceed 75 min when consuming 90% of *ad libitum* intake.^21^ During their respective permitted time windows, adequate food was provided to all groups. The nutrient composition and levels of feed were provided in Table S1. The feed intake and body weight were monitored on day 0, 14, and 29. The dynamic anterior vena cava blood was collected at 6-h intervals over a 24-h period, starting on the 26th day of the experiment. On day 30, the pigs were euthanized after an overnight fast at ZT0 (7:00). Fasting blood was collected and immediately stored at 4°C, then centrifuged within 30 min to minimize the proteolytic variability. The samples of serum were stored at −80°C for determination of hormones. The proximal colonic digesta was collected and stored at −80°C for metagenomics and metabolomics analyses. The tissues of hypothalamus and colon were collected and stored at −80°C for determination of gene and protein expressions.

### Behavior monitoring and definition

The behavior of pigs was determined by video camera for three days on day 21. Feeding behavior was defined as the head of animal being within feeder for least 5 s, or when the animal was chewing food or grazing with its mouth in contact with the food.

### Transcriptome analysis of hypothalamus

The hypothalamus total RNA was extracted using Trizol reagent following the manufacturer’s protocol. Subsequently, high-quality RNA samples were utilized for mRNA purification through Dynabeads Oligo (dT) (Thermo Fisher, CA, USA). The resulting mRNA was fragmented and reverse-transcribed into cDNA, which served as a template for synthesizing U-labeled second-stranded DNAs. After size selection and amplification, a final cDNA library with an average length of 300 ± 50 bp was constructed. Paired-end sequencing (PE150) was conducted on an Illumina Novaseq™ 6000 (LC-Bio Technology CO., Ltd., Hangzhou, China), adhering to the recommended protocol provided by the vendor. The clean data was obtained and aligned to the reference genome Ensembl_v107. The gene expression abundance was estimated by calculating the fragment per kilobase of transcript per million mapped reads (FPKM). The differentially expressed genes (DEGs) were identified using the criteria of *p* < 0.05 and |log_2_ (FC)| > 1, and enrichment analysis of Kyoto Encyclopedia of Genes and Genomes (KEGG) pathway was performed using the OmicStudio platform (https://www.omicstudio.cn/tool).

### Determination of gastrointestinal hormones

The hypothalamic grinding powder was dissolved in sterile saline and centrifuged to obtain the supernatant. The blood samples were centrifuged to obtain serum. Gastrointestinal hormones, including ghrelin, leptin, glucagon, insulin (INS), 5-hydroxytryptamine (5-HT), GLP-1, PYY, cholecystokinin (CCK), glucose-dependent insulinotropic polypeptide (GIP), gastrin and dopamine (DA), were quantified using ELISA kits following the manufacturer’s instructions (Nanjing MALLBIO Biological Technology Co.,Ltd, Nanjing China).

### Real-time quantitative PCR analysis

The total RNA of hypothalamic and colonic tissues was extracted using the EASYspin Plus RNA extraction kit (Aidlab, China). Following dilution, the RNA was reverse transcribed into cDNA using the *Evo M-MLV* Mix Kit (Accurate Biology, AG11728, China). Target gene expressions were determined using the SYBR Green Premix *Pro Taq* HS qPCR Kit (Rox Plus) (Accurate Biology, AG11718, China) and quantified by normalizing with the reference gene β-actin according to the formula 2^−ΔΔCt^. The primer sequences were provided in Table S2.

### Western blot analysis of colonic tissue

The colonic tissues were lysed in RIPA buffer (NCM Biotech, Suzhou, China) supplemented with protease inhibitors (Beyotime Biotech, Shanghai, China). The lysate was collected by centrifugation for gel electrophoresis. Following adjustment to the appropriate concentration, protein samples were separated using a 4–20% precast polyacrylamide gel electrophoresis (PAGE) gel and transferred onto polyvinylidene difluoride (PVDF) membranes. PVDF membranes were blocked using NcmBlot blocking buffer (NCM Biotech, Suzhou, China), followed by overnight incubation at 4°C with antibodies against ChgA (1:100 diluted; ABclonal, A9576), Notch1 (1:1000 dilution; ABclonal, A19090) and β-actin (1:2000 dilution; proteintech 20,536–1-AP). After washing steps, the membranes were further incubated with a secondary antibody (1:3000 dilution; ABclonal AS014) at room temperature. Finally, the blots were visualized using ChemiDoc Imager system (Bio-Rad) after immersion in enhanced chemiluminescence (ECL) HRP substrate solution (Yeasen Biotech, Shanghai, China). The ratio of target protein to β-actin was calculated using imageJ software (v 1.54 g).

### Immunofluorescence staining of colonic tissue

The colonic tissues were fixed with paraformaldehyde and subsequently embedded in wax blocks. Following sectioning and de-waxing, permeation was performed at room temperature using 0.5% Triton X-100 (Solarbio, Beijing, China). Tissue sections were then subjected to blocking and incubated with primary antibodies targeting Olfm4 (1:100 diluted; Cell Signaling Technology, #14369), Muc2 (1:100 diluted; ABclonal, A14659), and ChgA (1:100 diluted; ABclonal, A9576). After three washes, the tissue sections were incubated with secondary antibodies for 60 min at room temperature. DAPI was employed for nuclear staining, and fluorescence micrographs were acquired using the panoramic scan. The mean fluorescence intensity of Olfm4 and Muc2 protein staining were quantified using the “Threshold” feature in ImageJ software (v 1.54 g). The quantity of positive cells was determined using the “Analyzed Particles” feature in ImageJ software (v 1.54 g).

### DNA extraction and metagenomic sequencing

The total DNA of colonic digesta was extracted using the E.Z.N.A.® Viral DNA Kit (Omega Bio-tek, Norcross, GA, U.S.) following the manufacturer’s protocols. The TruSeq DNA Library Preparation kit of Illumina was employed to construct sequencing libraries using high-quality DNA samples. Clean reads were obtained after quality trimming using Trimmomatic^22^ and removal of host-genome contaminations using the Burrows−Wheeler Alignment Tool (http://bio-bwa.sourceforge.net/bwa.shtml). The contigs of each sample were generated from clean sequence reads using MegaHit.^23^ Prediction of the open reading frames (ORFs) of assembled contigs was performed using Prodigal v2.6.3, and clustering analysis was conducted using CD-HIT.^24^ The values of transcripts per kilobase of exon model per million mapped reads (TPM) were calculated to represent gene abundance. The species abundance and gene function were annotated using the non-redundant protein (NR) database and KEGG database. Linear discriminant analysis effect size (LEfSe) analysis was conducted to identify the characteristic species using the R microeco package (v 1.6.0).

### Analysis of colonic digesta metabolomics

For non-targeted metabolomics analysis, the colonic digesta sample was mixed with 80% methanol and subjected to ultrasound treatment. After incubation at −40°C for 1 h, the supernatant was obtained through high-speed centrifugation and supplemented with 2-Chlorophenylalanine for subsequent analysis. The determination of metabolites was performed using Liquid Chromatograph Mass Spectrometer (LC-MS) analysis with mobile phases consisting of water containing 0.05% ammonium and acetonitrile. Raw data was normalized by probability quotient normalization (PQN) with quality control (QC), log transformation and auto scaling. The SIMCA-p software (v 14.0, Umetrics, Umea, Sweden) was used to calculate variable importance projection (VIP) values. The identification of different metabolites was based on the criteria of VIP > 1 and *p* < 0.05 between the ALF pattern and three time-restricted feeding patterns.

For targeted metabolomics analysis, the colonic digesta was mixed with 80% methanol followed by grinding. The supernatant was collected after centrifugation and diluted with 10% methanol. The diluent was mixed with the 20 μg/L L-Tryptophan-d5 solution for subsequent LC-MS analysis. Standards including tryptophan, indole-3-lactic acid (ILA), indole-3-propionic acid (IPA), indole, tryptamine, IAA, indole-3-aldehyde (IAld), and 5-HT were obtained to quantify the concentrations of these metabolites.

### Microbial culture and metabolite measurements

*Ligilactobacillus salivarius* and *Ligilactobacillus animalis* stains isolated from pig gut were cultured in MRS liquid medium contained 1 mM tryptophan for 12 h. After filtration, the supernatant was mixed with a fourfold volume of methanol and placed in the −80°C refrigerator for 0.5 h. The supernatant was collected after centrifugation and then evaporated. Methanol was subsequently added for redissolution. The supernatant was taken for High performance liquid chromatography (HPLC) measurement after high-speed centrifugation. A total of 10 μL supernatant was injected and separated by gradient elution. The concentration of acetonitrile containing 0.1% formic acid (v/v) was varied from 5% to 95% over a period of 20 min at a flow rate of 0.8 mL/min, with detection performed at a wavelength of 280 nm.

### *Experimental design and sample collection for short-term* in vivo *validation*

To investigate the impact of ILA on GLP-1 secretion *in vivo*, a 7-day short-term experiment using weaned piglets was conducted. A total of 12 Duroc × Landrace × Large White pigs (7.41 ± 0.16 kg) were randomly assigned to two groups: the control group and ILA group (Figure 8a). Each group consisted of 6 replicates (pens) with 1 pig per pen. Pigs in the control group were fed a basal diet, while those in the ILA group received the same basal diet supplemented with 100 mg/kg of ILA. All pigs were provided with feed and water *ad libitum*. After an overnight fast, blood was collected from the anterior vena cava and stored at 4°C. Serum samples were obtained by centrifugation within 30 min for subsequent measurement of GLP-1 levels.

### Intestinal crypt isolation and intestinal organoid culture

The jejunal crypts were isolated from a 7-day-old piglet and cultured as previously described.^25^ A suitable segment of middle jejunum was longitudinally dissected, and the intestinal mucosa was removed using sterilized slides. Then, the jejunum was cut into 1 cm × 1 cm pieces. Throughout this process, cold PBS containing antibiotics was continuously rinsed until the supernatant remained clear. The tissue pieces were digested with 25 mL of Gentle Cell Dissociation Reagent (Stemcell) for 30 min at room temperature. The crypts were isolated by filtration through a 70 μm filter followed by centrifugation, and subsequently resuspended in PBS supplemented with 1% fetal bovine serum. After counting, the intestinal crypts were resuspended in IntestiCult organoid growth medium (Stemcell) and Matrigel (BD Biosciences), and planted onto a 24-well plate. Following gelation, the crypts were supplemented with 500 μL of organoid growth medium and incubated at 37°C with 5% CO_2_. After passaging, the intestinal organoids were treated with different concentrations of ILA for 60 h. The total RNA of intestinal organoids was extracted using the EASYspin Plus RNA extraction kit (Aidlab, China), and the medium supernatant was collected for GLP-1 determination using an ELISA kit (Nanjing MALLBIO Biological Technology Co., Ltd, Nanjing China). The expression levels of ChgA at the protein level were determined using immunofluorescence staining and western blot analysis. Briefly, the organoids were fixed using paraformaldehyde and permeabilized using 0.2% Triton X-100 (Solarbio, Beijing, China). After blocking, the organoids were incubated with primary antibodies targeting ChgA (1:100 diluted; ABclonal, A9576) overnight at 4°C. After washing, the organoids were incubated with the secondary antibody (1:250 diluted; Bioworld, BS10017) for 1 h at room temperature. After DAPI staining, the fluorescence of the organoids was identified using the panoramic scan. Additionally, the protein of organoids was extracted using RIPA buffer supplemented with protease inhibitors for subsequent western blot analysis.

### STC-1 cell culture and treatment

The intestinal secretin tumor cell line (STC-1) was cultured in Dulbecco’s modified eagle medium (DMEM, high glucose). ILA at concentrations of 10, 100, and 1000 μM was added to the medium for 6 h and 12 h. Diprotin A was added to the cell medium in order to prevent degradation of GLP-1. After incubation, the cell supernatant was collected for GLP-1 determination using an ELISA kit.

### Statistical analyses

The growth performance, hormone levels, metabolite levels, as well as gene and protein expressions from both *in vivo* and *in vitro* experiments were analyzed using one-way ANOVA followed by post-hoc Duncan’s test and Student’s t-test. The behavioral data following a normal distribution were subjected to one-way ANOVA analysis, while the behavioral data with a non-normal distribution were analyzed using the Kruskal-Wallis test. The microbiological data were assessed using the Kruskal-Wallis test.

## Results

### Time-restricted feeding patterns reduced feed intake and affected eating behavior

The average daily feed intake of ALF at 0–14 d exceeded that of the three time-restricted feeding groups, and surpassed both eTRF and mTRF at 14–29 d. During the final 15 days of the experiment, the average daily feed intake of TRF pattern was higher than eTRF and mTRF patterns (Figure 1b). Correspondingly, ALF resulted in a significant increase in body weight compared to the other groups on day 14, and further increased body weight compared to eTRF and mTRF on day 29. TRF increased body weight compared to eTRF and mTRF on day 29, but there was no significant difference between TRF and ALF (Figure 1c). Additionally, the feed/gain ratio of ALF was significantly higher than that of other groups at 14–29 d (Figure 1d). The analysis of eating behaviors revealed that ALF group increased the overall duration of eating compared to other groups (Figure 1e). The frequency of eating was significantly higher in the ALF pattern compared to the TRF and mTRF patterns (Figure 1f). No significant impact was observed on eating rate (Figure 1g). It is noteworthy that, based on the hourly statistics, the eating time of pigs with three time-restricted feeding patterns fell within the permissible time range for each restricted window (Figure 1h).

### Time-restricted feeding impacted appetite-related hormone dynamic levels and increased hypothalamic and serum GLP-1 levels

**Figure 2.**
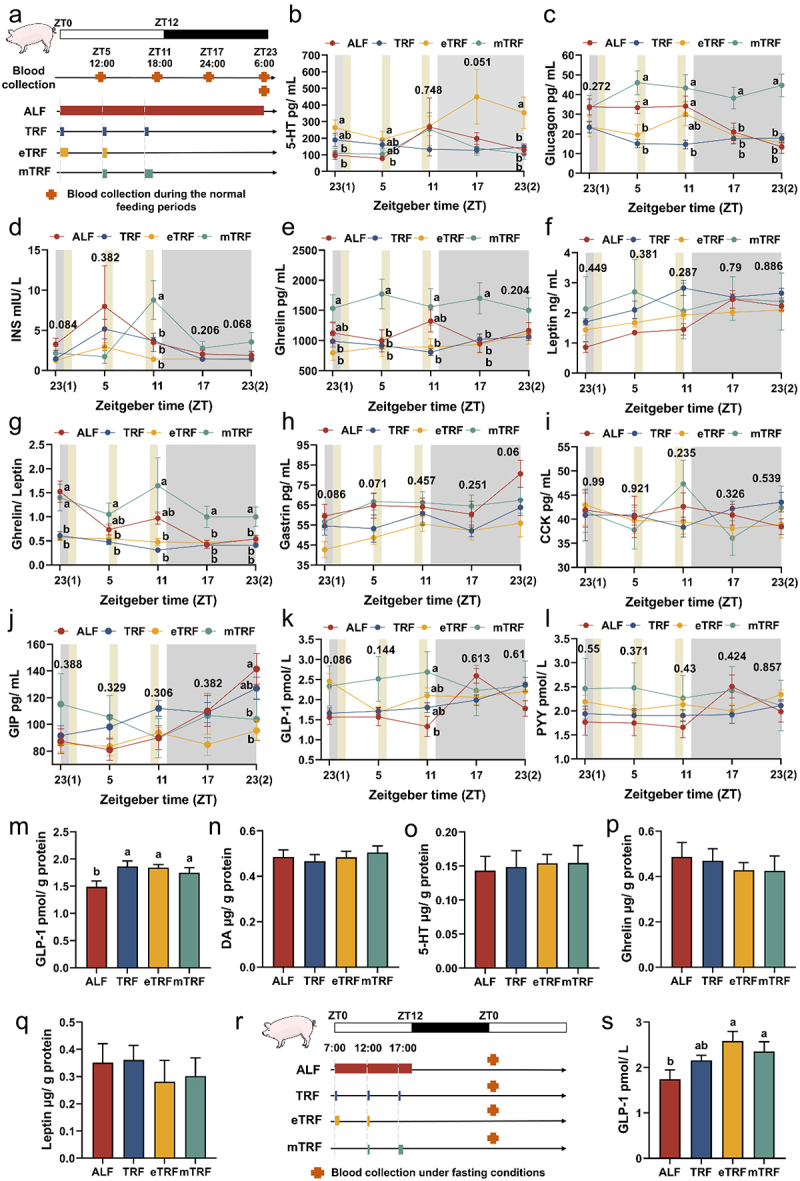
Effect of feeding patterns on the levels of neurotransmitter and gastrointestinal hormones. (a) Experimental procedure of dynamic serum collection during the normal feeding periods. Dynamic changes in serum levels of (b) 5-hydroxytryptamine (5-HT), (c) glucagon, (d) insulin (INS), (e) ghrelin, (f) leptin, (g) ghrelin/leptin ratio, (h) gastrin, (i) cholecystokinin (CCK), (j) glucose-dependent insulinotropic polypeptide (GIP), (k) glucagon-like peptide-1 (GLP-1) and (l) peptide YY (PYY) (*n* = 7). The gray shaded areas represent the dark-phase periods. The levels of (m) GLP-1, (n) DA, (o) 5-HT, (p) ghrelin, and (q) leptin in the hypothalamus (*n* = 7). (r) Experimental procedure of fasting serum collection. (s) The levels of GLP-1 in the fasting serum (*n* = 7). Data are presented as the mean ± SEM. Different letters denote statistically significant differences among the groups (*p* < 0.05).

**Figure 3.**
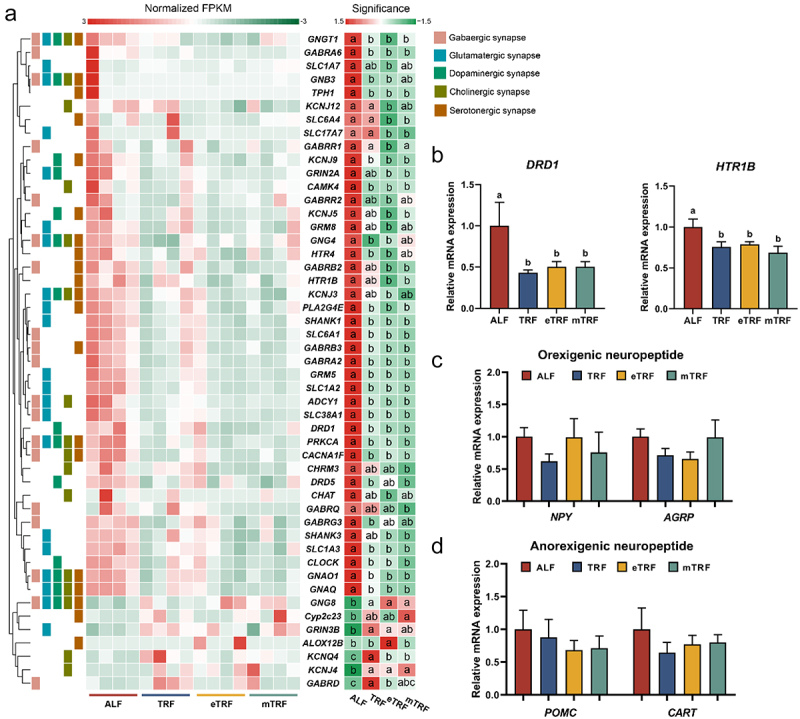
Effects of feeding patterns on hypothalamic transcription profile. (a) The gene expressions of differential gene associated with neurotransmitter pathways (*n* = 4). Significance denotes the statistically significant differences among various groups, as determined by one-way ANOVA followed by post-hoc Duncan’s test. (b) The mRNA expressions of *DRD1* and *HTR1B* (*n* = 7). (c) The mRNA expressions of orexigenic neuropeptides (*NPY* and *AGRP*) (*n* = 7). (d) The mRNA expressions of anorexigenic neuropeptides (*POMC* and *CART*) (*n* = 7). Data are presented as the mean ± SEM. Different letters denote statistically significant differences among the groups (*p* < 0.05).

The levels of neurotransmitter 5-HT and gastrointestinal hormones were quantified using dynamic serum samples collected every 6 h during the normal feeding periods, taking into account the influence of food intake timing and circadian clocks (Figure 2a). As shown in Figure 2b, the levels of appetite-related neurotransmitter 5-HT in the eTRF pattern were significantly elevated compared to those in the ALF pattern at ZT23 (1), ZT5 and ZT23(2). The levels of glucagon in the mTRF pattern were consistently elevated across four time points, exhibiting significant increases compared to eTRF and TRF at ZT5, significant increases compared to TRF at ZT11, and significant elevations compared to TRF, eTRF, and mTRF at both ZT17 and ZT23(2) (Figure 2c). However, INS levels were higher in the mTRF group compared to the other three groups at ZT11 (Figure 2d). Higher levels of ghrelin and the ghrelin-to-leptin ratio in the mTRF group were observed, except for ghrelin at ZT23(2) and the ghrelin-to-leptin ratio at ZT23(1), despite there being no significant differences in leptin levels among the four groups across 24 h (Figure 2e-g). The levels of dynamic gastrin, CCK, and PYY showed no significant differences over a 24-h period (Figure 2h,i and l). However, at ZT23 (2), the GIP levels of ALF were found to be higher compared to mTRF and eTRF (Figure 2j). The levels of GLP-1 in ALF group were consistently low, except at ZT17, indicating a noticeable trend at ZT23 (1) and a significant difference at ZT11 (Figure 2k). To investigate the potential associations between neurotransmitters and hormones with central regulation of appetite, the levels of GLP-1, DA, 5-HT, ghrelin, and leptin in hypothalamic tissue were determined. The results revealed a significant decrease in GLP-1 levels in the ALF pattern compared to those on time-restricted feeding patterns (Figure 2m). However, the hypothalamic levels of DA, 5-HT, ghrelin and leptin exhibited no significant differences (Figure 3n–q). Furthermore, fasting serum GLP-1 levels exhibited a significant decrease in the ALF pattern compared to eTRF and mTRF patterns (Figure 3r,s).

### Time-restricted feeding patterns altered hypothalamic transcription profile

Transcriptomic analysis of the hypothalamus, a pivotal feeding center, revealed that ALF significantly upregulated 481, 856, and 1011 gene expressions and downregulated 674, 856, and 1279 gene expressions compared to TRF, eTRF and mTRF, respectively (Figure S1A, S2A and S3A). Interestingly, the DEGs between ALF pattern and the three time-restricted feeding patterns were found to be significantly enriched in neurotransmitter pathways, including GABAergic synapse, glutamatergic synapse, cholinergic synapse, serotonergic synapse and dopaminergic synapse (Figure S1B, S2B and S3B). ALF resulted in the upregulation of a majority of gene expressions that were enriched in neurotransmitter pathways (Figure 3a and Figure S1C, S2C and S3C). Notably, ALF upregulated the expressions of *DRD1*, *DRD5*, *HTR4* and *HTR1B*. Furthermore, real-time PCR analysis demonstrated higher gene expressions for *DRD1* and *HTR1B* in the ALF pattern compared to the three time-restricted patterns (Figure 3b). However, no statistically significant differences were observed in the expressions of *NPY*, *AGRP*, *POMC* and *CART* (Figure 3c,d).

### The eTRF pattern induced enteroendocrine cell differentiation

**Figure 4.**
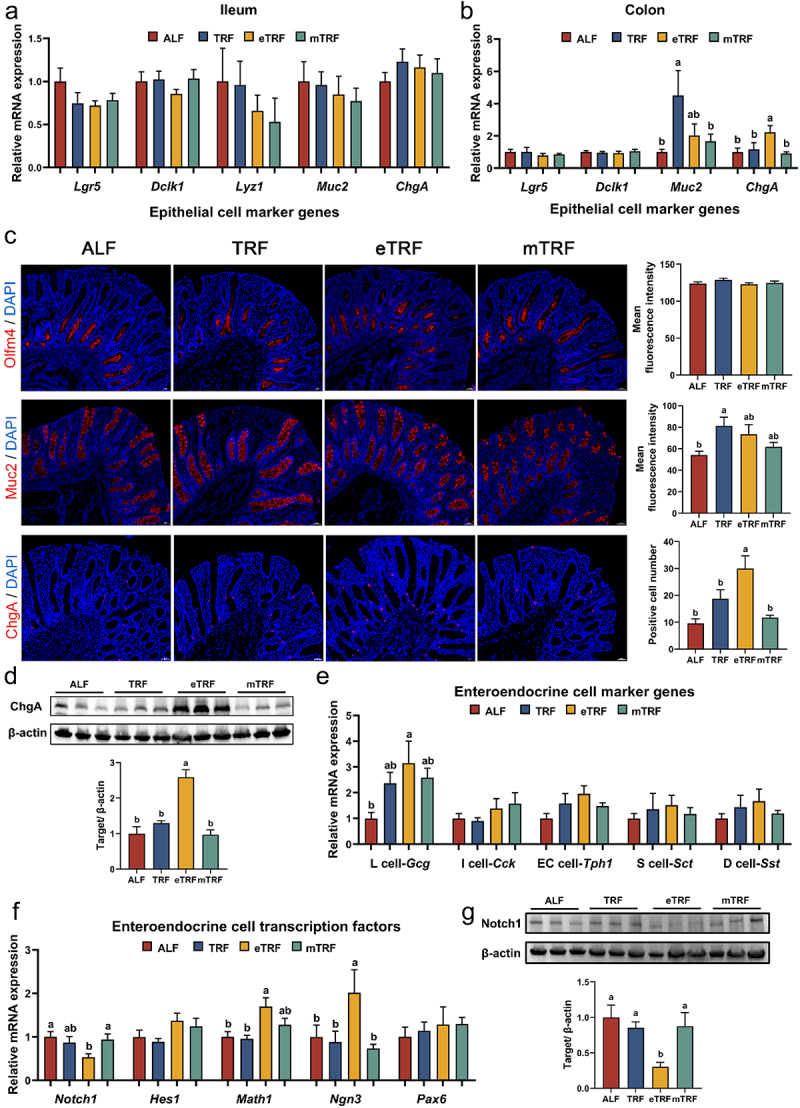
Effect of feeding patterns on intestinal stem cell proliferation and differentiation. (a) The mRNA expressions of epithelial cell marker genes in the ileum (*n* = 7). (b) The mRNA expressions of epithelial cell marker genes in the colon (*n* = 7). (c) Immunofluorescence staining of Olfm4, Muc2 and ChgA in pig colonic tissue (*n* = 6). (d) The protein expression of ChgA (*n* = 3) in the colon. (e) The mRNA expressions of enteroendocrine cell marker genes (*n* = 7) in the colon. (f) The mRNA expressions of enteroendocrine cell transcription factors (*n* = 7) in the colon. (g) The protein expression of Notch1 (*n* = 3) in the colon. Data are presented as the mean ± SEM. Different letters denote statistically significant differences among the groups (*p* < 0.05).

To elucidate the underlying factors contributing to elevated GLP-1 levels under time-restricted feeding, the expression levels of genes involved in stem cell proliferation and differentiation in the ileum and colon were assessed. The results demonstrated that feeding patterns had no significant impact on the expression levels of markers related to stem cell proliferation and differentiation in the ileum (Figure 4a). Additionally, the expressions of active intestinal stem cell marker (*Lgr5*) and tuft cell marker (*Dclk1*) showed no significant difference in the colon, as shown in Figure 4b. And no significant difference was observed in the mean fluorescence intensity of the active intestinal stem cell marker Olfm4 in the colon under different feeding patterns (Figure 4c). However, compared to ALF and mTRF, TRF significantly upregulated the expression of goblet cell marker gene (*Muc2*) in the colon. Additionally, eTRF exhibited an upregulation in the expression of EEC marker gene (*ChgA*) compared to ALF, TRF, and mTRF in the colon (Figure 4b). Meanwhile, the immunofluorescent staining of colonic tissues revealed that the mean fluorescence intensity of Muc2 was higher in the TRF pattern compared to the ALF pattern, and the number of EECs increased in the eTRF pattern compared to the ALF, TRF, and mTRF patterns. (Figure 4c). The results of the western blot analysis also demonstrated that eTRF significantly increased ChgA protein expression compared to the other groups (Figure 4d). Further investigations into EEC marker genes revealed that eTRF resulted in the upregulation of the L cell marker gene *Gcg* compared to ALF (Figure 4e). Consistently, among the three groups of time-restricted feeding, eTRF exhibited a significant downregulation of Notch1 gene and protein expressions (Figure 4f,g), as well as upregulation of gene expressions of *Math1* and *Ngn3* compared to ALF (Figure 4f).

### *The eTRF pattern facilitated the colonization of* Lactobacillus *and elevated the level of ILA in colonic digesta*

**Figure 5.**
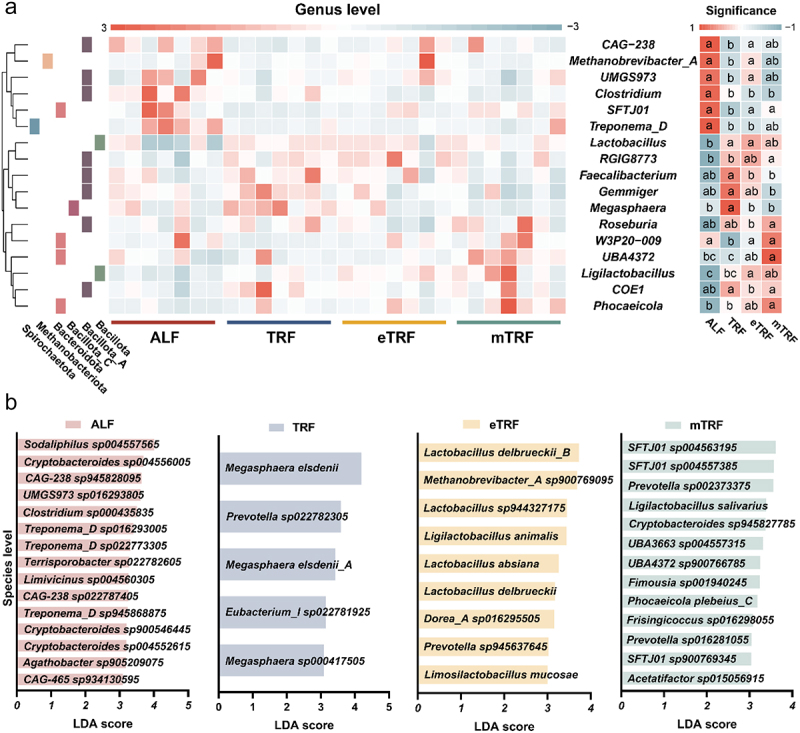
Effect of feeding patterns on colonic microbiota of pigs. (a) The relative abundance of significantly changed genera (relative abundance > 1%) (*n* = 7). The relative abundance at the genus level was scaled by row. Significance denotes the statistically significant differences among various groups, as determined by Kruskal-Wallis test. (b) Linear discriminant analysis (LDA) score plot of the featured microbial species in pig colonic microbiomes (LDA score > 3, *p* < 0.05). Different letters denote statistically significant differences among the groups (*p* < 0.05).

In order to investigate the potential microbiological mechanisms underlying the induction of EEC differentiation by feeding patterns, metagenomic sequencing was conducted to determine microbial composition and metabolic functions. The results shown in Figure 5a revealed that 17 genera exhibited statistically significant differences among the genera with relative abundance exceeding 1%. It is noteworthy that three time-restricted feeding patterns facilitated the colonization of *Lactobacillus* and *Ligilactobacillus*. Furthermore, TRF significantly facilitated the colonization of *Faecalibacterium* and *Megasphaera*, while both TRF and mTRF promoted the colonization of *Roseburia*. At the species level, LEfse analysis revealed that eTRF was predominantly characterized by *Lactobacillus*, including *Ligilactobacillus animalis*, *Lactobacillus absiana*, *Lactobacillus delbrueckii*, and *Limosilactobacillus mucosae*. TRF was primarily associated with bacteria involved in carbohydrate metabolism, such as *Megasphaera elsdenii* (Figure 5b).

**Figure 6.**
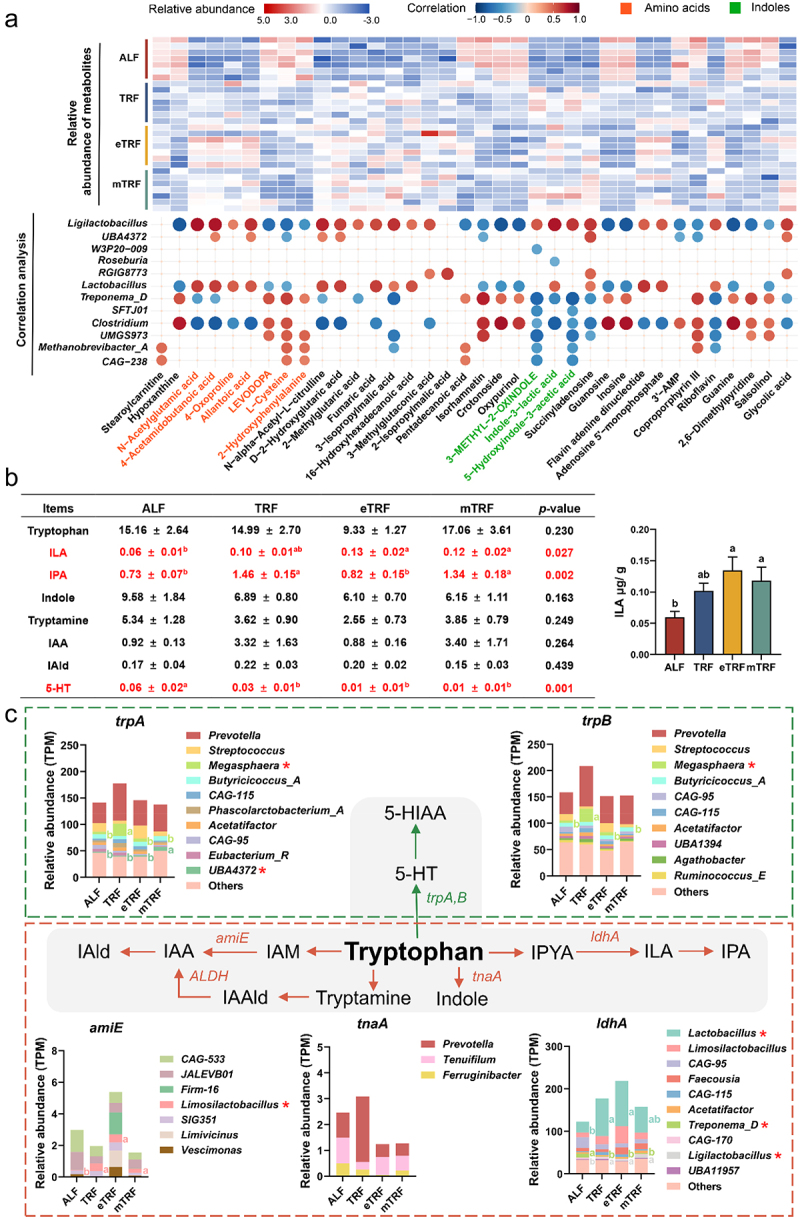
Metabolic reconstruction of tryptophan metabolism according to colonic metagenomic and metabolomic information. (a) The correlation analysis between the differential genera and metabolites in the colonic digesta. The raw data, after normalization with quality control (QC), log transformation, and auto-scaling, were standardized to represent the relative abundance of metabolites. (b) Concentrations (μg/g) of tryptophan metabolites in colonic digesta (*n* = 7). Data are presented as the mean ± SEM. Red represents metabolites with significant differences. (b) Tryptophan metabolism pathway in colonic microbes. The * denotes the differential genera that contribute genes. Data are presented as the mean. Different letters denote statistical differences in the gene abundance contributed by genera among the groups (*p* < 0.05). ILA, indole-3-lactic acid; IPA, indole-3-propionic acid; IAM, indole-3-acetamide; IAA, indole-3-acetic acid; IAld, indole-3-aldehyde; 5-HT, 5-hydroxytryptamine; 5-HIAA, 5-hydroxyindole-3-acetic acid.

To investigate the potential mechanisms underlying the difference in GLP-1 levels between the ALF pattern and the three time-restricted feeding patterns, an untargeted metabolomic analysis was conducted to identify possible intermediates involved in host-gut microbial interactions. As shown in Figure 6a, the correlation analysis between different genera and metabolites showed that *Lactobacillus* and *Ligilactobacillus* were positively correlated with indole metabolites, while negatively correlated with amino acids such as levodopa and cysteine. Thereinto, ILA, as a metabolite from tryptophan by intestinal microbes, was positively related to *Lactobacillus* and *Ligilactobacillus*. Targeted metabolomics analysis was conducted to quantify the concentrations of tryptophan metabolites in colonic digesta. The results demonstrated that eTRF and mTRF significantly elevated ILA levels compared to ALF. Additionally, TRF and mTRF showed higher concentrations of IPA relative to ALF and eTRF. Conversely, ALF led to a significant increase in 5-HT levels (Figure 6b). Based on metagenomic information, the metabolic pathway of tryptophan metabolism was elucidated. As shown in Figure 6c, eTRF exhibited a higher abundance of genes involved in the conversion of tryptophan into ILA and IAA. Notably, the key gene *ldhA* responsible for the conversion of tryptophan into ILA primarily contributed by *Lactobacillus* and *Ligilactobacillus*.^26^ And *Limosilactobacillus* primarily contributed to the key gene *amiE* responsible for the conversion of tryptophan into IAA.

**Figure 7.**
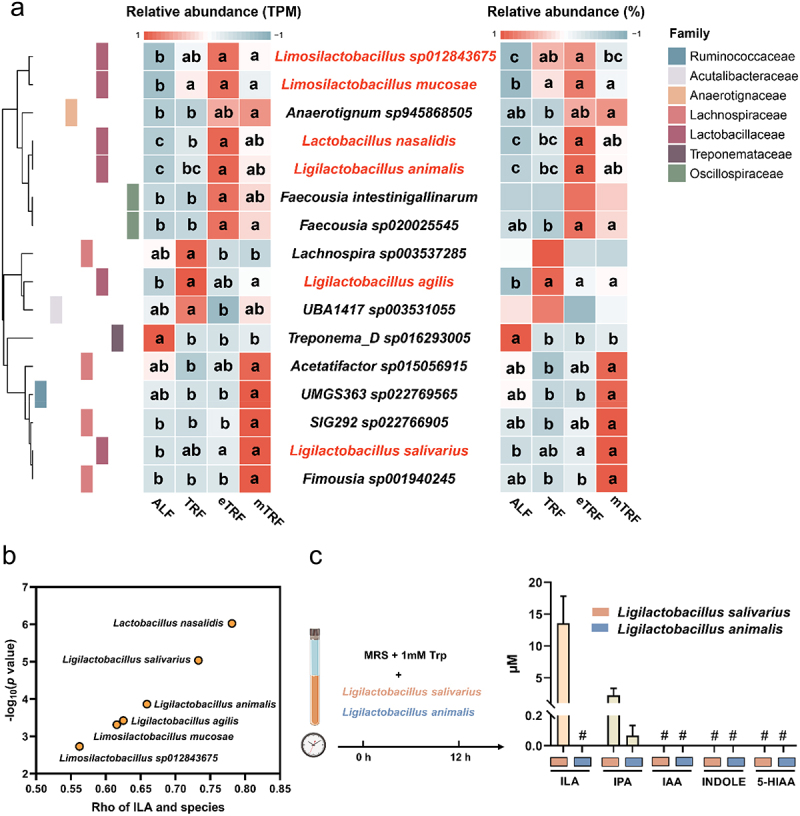
Confirmation of the key bacteria that convert tryptophan to ILA. (a) Phylogenetic distribution analysis. (b) Correlation analysis of species and ILA. (c) High performance liquid chromatography (HPLC) measurement (*n* = 3). The # means metabolites were not detected. Data are presented as the mean ± SEM. Different letters denote statistically significant differences among the groups (*p* < 0.05). Trp, tryptophan.

The species assigned by *ldhA* were shown in Figure 7a. The relative abundance of *ldhA* contributed by *Limosilactobacillus sp012843675*, *Limosilactobacillus mucosae*, and *Ligilactobacillus salivarius* in the eTRF pattern exhibited a significantly higher levels compared to that observed in the ALF pattern. And the eTRF showed a significantly higher abundance of *ldhA* contributed by *Lactobacillus nasalidis* and *Ligilactobacillus animalis* compared to the TRF and ALF patterns. Correlation analysis showed that these species were positively correlated with ILA levels in colonic digesta. Among them, the correlation coefficients of *Lactobacillus nasalidis*, *Ligilactobacillus salivarius*, and *Ligilactobacillus animalis* were higher (Figure 7b). To validate the tryptophan conversion capability, *Ligilactobacillus salivarius* and *Ligilactobacillus animalis* were cultured in MRS medium supplemented with 1 mM tryptophan. As shown in Figure 7c, ILA and IPA were identified in the supernatant of *Ligilactobacillus salivarius*, while IPA was also detected in the supernatant of *Ligilactobacillus animalis*.

### ILA increased serum GLP-1 levels and facilitated the differentiation of intestinal stem cells into enteroendocrine cells

**Figure 8.**
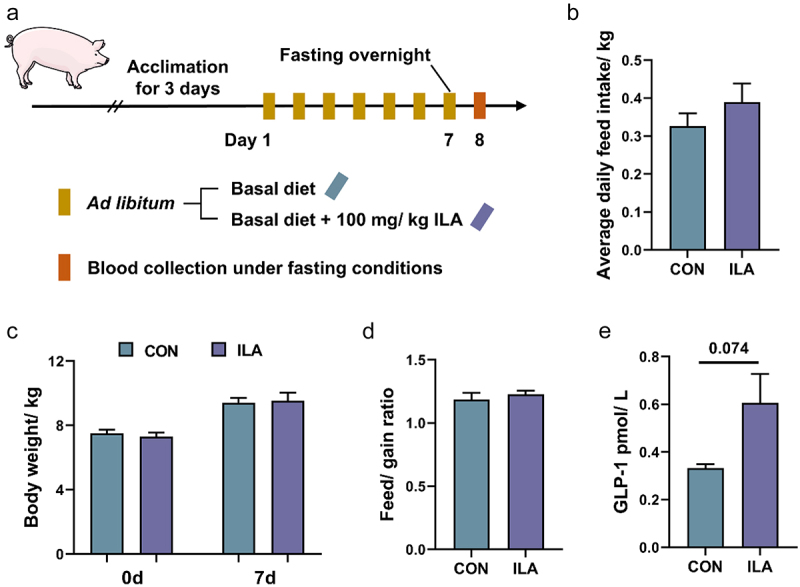
Effects of ILA on growth performance and serum GLP-1 levels. (a) Experimental procedure. (b) Average daily feed intake (*n* = 6). (c) Body weight (*n* = 6). (d) The ratio of feed and gain (*n* = 6). (e) The levels of GLP-1 in the fasting serum (*n* = 6). Data are presented as the mean ± SEM.

**Figure 9.**
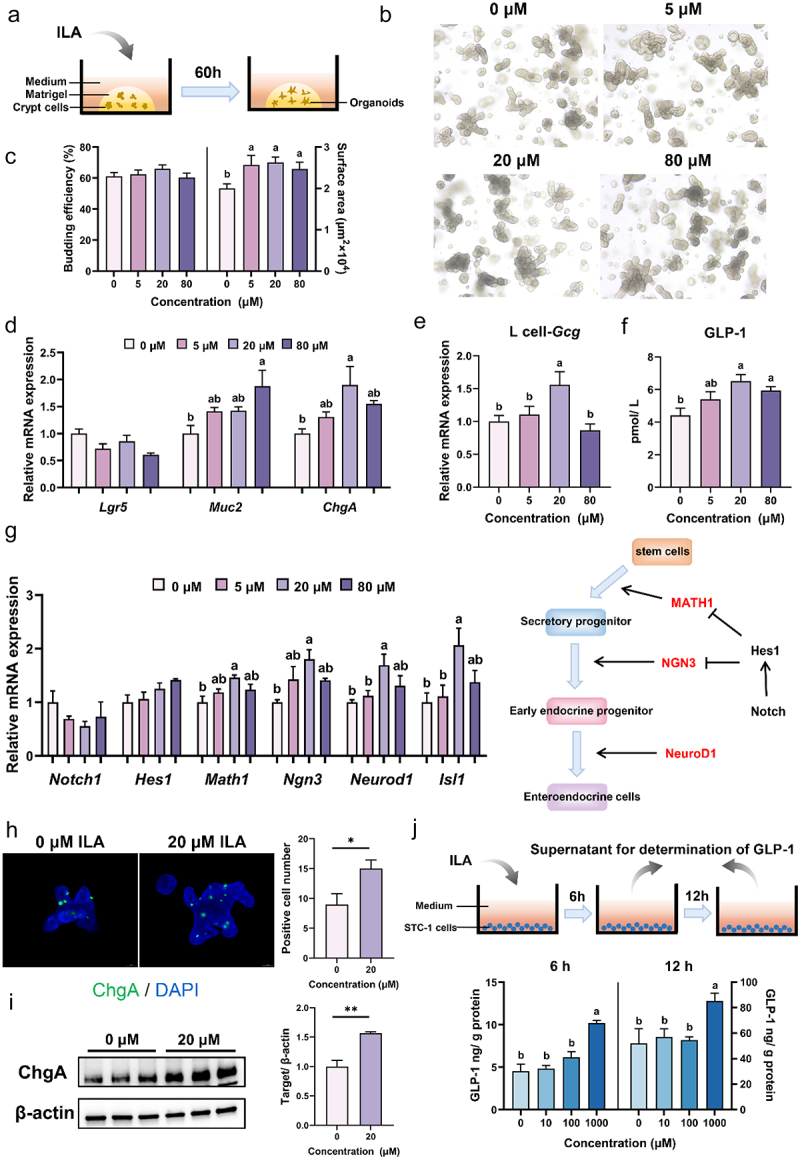
Effects of ILA on GLP-1 secretion in intestinal organoids and STC-1 cells. (a) Schematic of the pig intestinal organoid experiments. (b) The representative images of intestinal organoid morphology (*n* = 3). (c) Budding efficiency and surface area of organoids (*n* = 3). (d) The mRNA expressions of *Lgr5*, *Muc2* and *ChgA* (*n* = 3). (e) The mRNA expressions of *Gcg* (*n* = 3). (f) The GLP-1 levels in the organoid medium (*n* = 4). (g) The mRNA expressions of *Notch1*, *Hes1*, *Math1*, *Ngn3*, *Neurod1* and *Isl1* as well as the schematic of enteroendocrine cell (EEC) differentiation (*n* = 3). (h) Immunofluorescence staining of ChgA in organoids (*n* = 4). The * denotes *p* < 0.05. (i) The protein expression of ChgA in organoids (*n* = 3). The ** denotes *p* < 0.01. (j) Schematic of the STC-1 cell experiments and the GLP-1 levels in the cell medium (*n* = 3). Data are presented as the mean ± SEM. Different letters denote statistically significant differences among the groups (*p* < 0.05).

To further investigate the impact of ILA on GLP-1 secretion *in vivo*, a short-term 7-day experiment was conducted (Figure 8a). Although no significant difference was observed in the average daily feed intake, body weight, and feed-to-gain ratio (Figure 8b–d), supplementation with ILA tended to increase serum GLP-1 levels (Figure 8e). To investigate whether microbial metabolite ILA play a pivotal role in EEC differentiation, jejunal crypts were isolated from a 7-day-old pig and cultured with different concentrations of ILA for a duration of 60 h (Figure 9a). The results indicated that exposure to 5 μM, 20 μM and 80 μM ILA led to an increase in the surface area of the intestinal organoids, whereas the budding efficiency remained unchanged compared to the mock-treated group (Figure 9b,c). As shown in Figure 9d, 80 μM ILA significantly increased the gene expressions of the goblet cell marker (*Muc2*) and 20 μM ILA significantly increased the expressions of the EEC marker gene (*ChgA*) compared to the mock-treated group. Additionally, treatment with 20 μM ILA resulted in the upregulation of *Gcg* gene expressions and an increase in GLP-1 levels within the supernatant of the organoid culture medium (Figure 9e,f). Moreover, 20 μM ILA significantly upregulated the expressions of *Math1*, *Ngn3*, *Neurod1* and *Isl1* (Figure 9g). The results of the immunofluorescence staining and western blot analysis demonstrated that 20 μM ILA significantly increased the number of ChgA-positive cells (Figure 9h) and the protein expression levels of ChgA (Figure 9i). In order to investigate the potential of ILA in promoting GLP-1 secretion in intestinal EECs, STC-1 cells were treated with ILA for 6 h and 12 h, and the levels of GLP-1 in the medium were determined. The results showed that only treatment with 1000 μM ILA significantly enhanced the secretion of GLP-1 by STC-1 cells (Figure 9J).

## Discussion

Numerous clinical trials have revealed that time-restricted feeding can regulate appetite, thereby avoiding the health burden of consuming food at inappropriate times.^6^ However, the underlying mechanism involved in appetite regulation of time-restricted feeding remains inadequately investigated. In this study, the pig model was used to replicate four prevalent eating habits to elucidate the microbiological mechanisms of appetite regulation.

In this study, the monitoring of feed intake and eating behaviors revealed that three time-restricted feeding patterns decreased food consumption while still meeting the growth requirements of pigs. Meanwhile, ALF upregulated the expressions of DA receptors (*DRD1* and *DRD5*) and 5-HT receptors (*HTR4* and *HTR1B*) compared to the time-restricted feeding patterns. The mechanisms of appetite control include homeostatic feeding circuits and reward-related circuits. In brief, the hypothalamus integrates peripheral signals of energy deficit and modulates POMC and NPY/AgRP neuronal activity to meet physiological needs, while DA signaling contributes to appetite control by serving as an incentive and motivational factor.^27^ The signaling of DA neurons exerts an orexigenic effect by directly inhibiting POMC neurons and exciting AGRP neurons in the hypothalamic arcuate nucleus (ARC), which is associated with hedonic or motivational aspects related to feeding.^28^ The activation of NPY-expressing DRD1 neurons in the ARC rapidly induces voracious feeding in mice within 1 h, without exerting a long-lasting effect after 24 h. In the case of inhibiting AGRP and NPY neurons in ARC, the activation of DRD1 neurons does not significantly impact short-term (4 h) food consumption, but it increases long-term (24 h) food intake. This underscores the role of DRD1 neurons in stimulating sustained feeding.^29^ In addition, although the upregulation of 5-HT signaling is generally thought to suppress appetite, a counter-regulatory response in the hypothalamic hedonic system leads to an upregulation of elements in the 5-HT signaling pathway.^30^ The upregulation of *HTR4* and *HTR1B* by ALF may function as a negative feedback mechanism within the reward pathway. Importantly, lower levels of hypothalamic and serum GLP-1 were found in the ALF pattern. GLP-1, an anorexigenic hormone secreted by L cells in the distal intestine, exhibits the ability to traverse the blood-brain barrier and modulate appetite through activation of anorexic neuropeptides or regulation of gastric emptying and intestinal motility.^31^ It is worth noting that GLP-1 was reported to affect the reward centers of brain to regulate feeding behavior. The activation of GLP-1 receptor through the GLP-1 agonist exenatide resulted in the suppression of synaptic strength onto ventral tegmental area (VTA) DA neurons projecting to the medial shell of nucleus accumbens (NAc), indicating that GLP-1 weakens the dopamine signaling on the projection from VTA to NAc.^11^ Exenatide reduced the cocaine-induced increase in synaptic DA levels by enhancing the expression of DA transporter on neuronal cell surfaces.^32^ Taken together, GLP-1 may play a role in inhibiting the hedonic system, which could contribute to the reduction in food intake associated with time-restricted feeding. Here, a pair-fed control group with calorie restriction was not established. Although the potential impact of feed intake differences on host metabolism cannot be avoided, the impact of different feeding patterns on the appetite of pigs can be reflected through differences in feed intake. The monitoring of eating time and body weight indicated that pigs subjected to a restricted feeding schedule for 29 days did not exhibit a negative energy balance. It is important to note that the reduced feed intake resulting from time-restricted feeding could also contribute to its beneficial effects on metabolic health.

As the largest endocrine system in the body, the gastrointestinal tract relies on hormone secretion from EECs to coordinate food intake, energy utilization, and nutrient absorption, thereby maintaining metabolic balance in the host.^13^ Furthermore, the differentiation of intestinal stem cells is crucial for the secretion of gastrointestinal hormones. And whether time-restricted feeding impacts GLP-1 secretion by influencing intestinal stem cell proliferation and differentiation remains incompletely understood. The results revealed that TRF upregulated goblet cell marker (Muc2), while eTRF upregulated EEC marker (ChgA) and L-cell marker (*Gcg*), indicating potential effects of different feeding patterns on intestinal stem cell fate. The EECs originate from multipotential progenitor cells located in the intestinal crypts.^12^ These cells are sparsely distributed throughout the mucosal layer, constituting approximately 1% of the total intestinal cells.^33^ The Notch signaling plays a crucial role in regulating the EEC differentiation by suppressing the expression of basic helix-loop-helix (bHLH) transcription factors, such as Math1, Ngn3, and Neurod1.^15^ Math1 is responsible for the development of precursor cells into three secretory cell types, including goblet, paneth, and enteroendocrine cells.^34^ Ngn3 is a downstream target of Math1 that controls endocrine differentiation.^35^ Neurod1 is a downstream target of Ngn3 that controls the terminal differentiation of S and I cell.^13^ In this study, eTRF upregulated the gene expressions of *Math1* and *Ngn3* and downregulated the gene and protein expression of Notch1 compared to other groups, suggesting enhanced EEC differentiation. Nevertheless, the potential mechanisms by which feeding patterns influence the secretion of endogenous GLP-1 within the gastrointestinal tract remain to be elucidated.

Gut microbiota and metabolites were reported to impact the EEC differentiation and gastrointestinal hormone secretion.^31^ Metagenomic and metabolomic analysis showed that three time-restricted feeding enhanced the colonization of *Lactobacillus* and increased the levels of the *Lactobacillus* metabolite ILA. These findings led to the hypothesis that time-restricted feeding may modulate gut microbiota and metabolites, thereby mediating EEC differentiation and GLP-1 secretion. To test this hypothesis, a short-term trial was conducted using weaned piglets to investigate whether ILA can increase serum GLP-1 levels. The results showed that supplementation with ILA for 7 days exhibited an increasing trend in serum GLP-1 levels. GLP-1 exhibited greater stability in *ex vivo* whole blood samples compared to *in vivo* circulation, and demonstrated higher stability in isolated plasma samples relative to whole blood samples.^36^ Therefore, the previous method was used to detect the serum GLP-1 levels,^37^ and low-temperature storage and rapid centrifugation ensured stabilization of GLP-1. Here, the level of GLP-1 detected was lower compared to that observed in the time-restricted feeding experiment, which may be attributed to the shorter duration of ILA supplementation and the younger age of the pigs. Higher levels of GLP-1 were observed in previous studies involving 110-day-old pigs.^37^

Previous studies have demonstrated that ILA can mitigate colitis in mice experiments and promote the proliferation of intestinal epithelial and progenitor cells to replenish damaged cells, as evidenced by organoid cultures.^38–40^ However, the impact of ILA on intestinal stem cell fate has not been well explored. Therefore, the pig intestinal organoid model was employed to investigate the impact of ILA on EEC differentiation. The results revealed that ILA could induce high levels of GLP-1 by modulating the differentiation of intestinal stem cells into EECs. This effect may be mediated through the upregulation of *Math1*, *Ngn3*, and *Neurod1* expressions. Progenitor cells undergo lineage commitment toward either a secretory or absorptive fate through the regulation of Hes1 and Math1. Notch signaling regulates Hes1 expression, thereby upregulating Math1 expression in progenitor cells and facilitating the commitment to a secretory fate. The upregulation of Hes1 expression leads to an increase in the number of proliferating cells.^41^ Deletion of Math1 results in all epithelial cells acquiring the characteristics of intact absorptive enterocytes.^42^ Although the Notch signaling pathway plays a pivotal role in early cell fate determination, it is important to consider that additional factors may also affect stem cell differentiation. For instance, Nrf2 has the potential to directly repress Math1 expression independent of any alterations in Hes1 expression.^43^ This may account for the lack of significant difference in Notch1 expression. In addition, the average concentration of ILA in the colonic digesta of pigs in the eTRF group was 0.13 μg/g, whereas 20 μM ILA promoted the differentiation of EECs *in vitro*. The ILA dosage gap may be attributed to variations in the duration of ILA action. In the 29-day time-restricted feeding experiment, ILA exerts prolonged effects on the intestine *in vivo*, whereas the influence of ILA on organoids is limited to 60 h *in vitro*. In addition, intestinal crypt cells were isolated from a 7-day-old pig, while the pigs used for the time-restricted feeding experiments were 50 days old. Age differences could potentially contribute as one of the factors. However, the required concentration (1000 μM) to promote GLP-1 secretion in STC-1 cells is much higher than the detected physiological ILA levels, which implied that ILA failed to stimulate GLP-1 secretion at physiological levels. In light of these findings, time-restricted feeding augmented GLP-1 levels by promoting the levels of *Lactobacillus* metabolite ILA, which was observed to facilitate the differentiation of intestinal stem cells into EECs rather than relying on EEC-mediated GLP-1 secretion. It is worth noting that other tryptophan metabolites, such as indole and IAA, may also contribute to promoting GLP-1 secretion. Previous studies have demonstrated their potential in enhancing GLP-1 release.^16,44^ Further research is required to elucidate the specific roles of these metabolites. Here, this study of time-restricted feeding lasted for 29 days. Although the prolonged effects after time-restricted feeding were not further researched, it was reported that the effects of changing eating windows on metabolic phenotype can persist after switching to *ad libitum* feeding.^45^ Furthermore, our previous research demonstrated that 29-d of *ad libitum* feeding led to the accumulation of liver fat and a significant elevation in hepatic function markers compared to time-restricted feeding.^46^ Therefore, the metabolic burden imposed by 4 weeks of inappropriate eating patterns may have long-lasting effects.

## Conclusion

This study investigated the potential microbiological mechanisms of time-restricted feeding in GLP-1 secretion and appetite regulation. Time-restricted feeding exerted a significant impact on long-term appetite regulation, characterized by reduced food intake and eating duration, elevated serum and hypothalamic GLP-1 levels, as well as suppressed reward-related circuits in the hypothalamus. This effect potentially resulted from the colonization of *Lactobacillus* and the higher levels of *Lactobacillus* metabolite ILA induced by time-restricted feeding. Supplementation with ILA was determined to increase fasting serum GLP-1 levels *in vivo* and facilitate the differentiation of intestinal stem cells into EECs in an organoid model. In summary, time-restricted feeding facilitated the secretion of GLP-1 and induced long-term suppression of appetite by modulating *Lactobacillus* colonization and microbial tryptophan metabolism. These findings shed light on the intricate interplay between eating patterns and appetite regulation.

## Supplementary Material

Supplemental Material

## Data Availability

The RNA-Seq transcriptome datasets are available at NCBI project PRJNA1151847. The metagenome sequencing data are available at NCBI project PRJNA1177368.
