# Supplementary material for: Time-restricted feeding promotes glucagon-like peptide-1 secretion and regulates appetite via tryptophan metabolism of gut Lactobacillus in pigs
Source: Gut Microbes. 2025 Feb 14;17(1):2467185. doi: 10.1080/19490976.2025.2467185 (PMC11834429; doi:10.1080/19490976.2025.2467185)

**Figure S1.** Differential analysis of hypothalamic transcriptome between the ALF pattern and the TRF pattern. (A) Volcano plots of DEGs. (B) The top 40 KEGG enrichment pathways. (C) The number of DEGs involved in neurotransmitter pathways.

**Figure S2.** Differential analysis of hypothalamic transcriptome between the ALF pattern and the eTRF pattern. (A) Volcano plots of DEGs. (B) The top 40 KEGG enrichment pathways. (C) The number of DEGs involved in neurotransmitter pathways.

**Figure S3.** Differential analysis of hypothalamic transcriptome between the ALF pattern and the mTRF pattern. (A) Volcano plots of DEGs. (B) The top 40 KEGG enrichment pathways. (C) The number of DEGs involved in neurotransmitter pathways.

**Figure S4.** Effects of feeding patterns on colonic microbiome. (A) Alpha diversity indices (Shannon and Simpson) at the genus level (n = 7). (B) Alpha diversity indices (Shannon and Simpson) at the species level (n = 7). (C) The top10 genera in different feeding patterns. Different letters denote statistically significant differences among the groups (*p* < 0.05).

**Figure S1.**


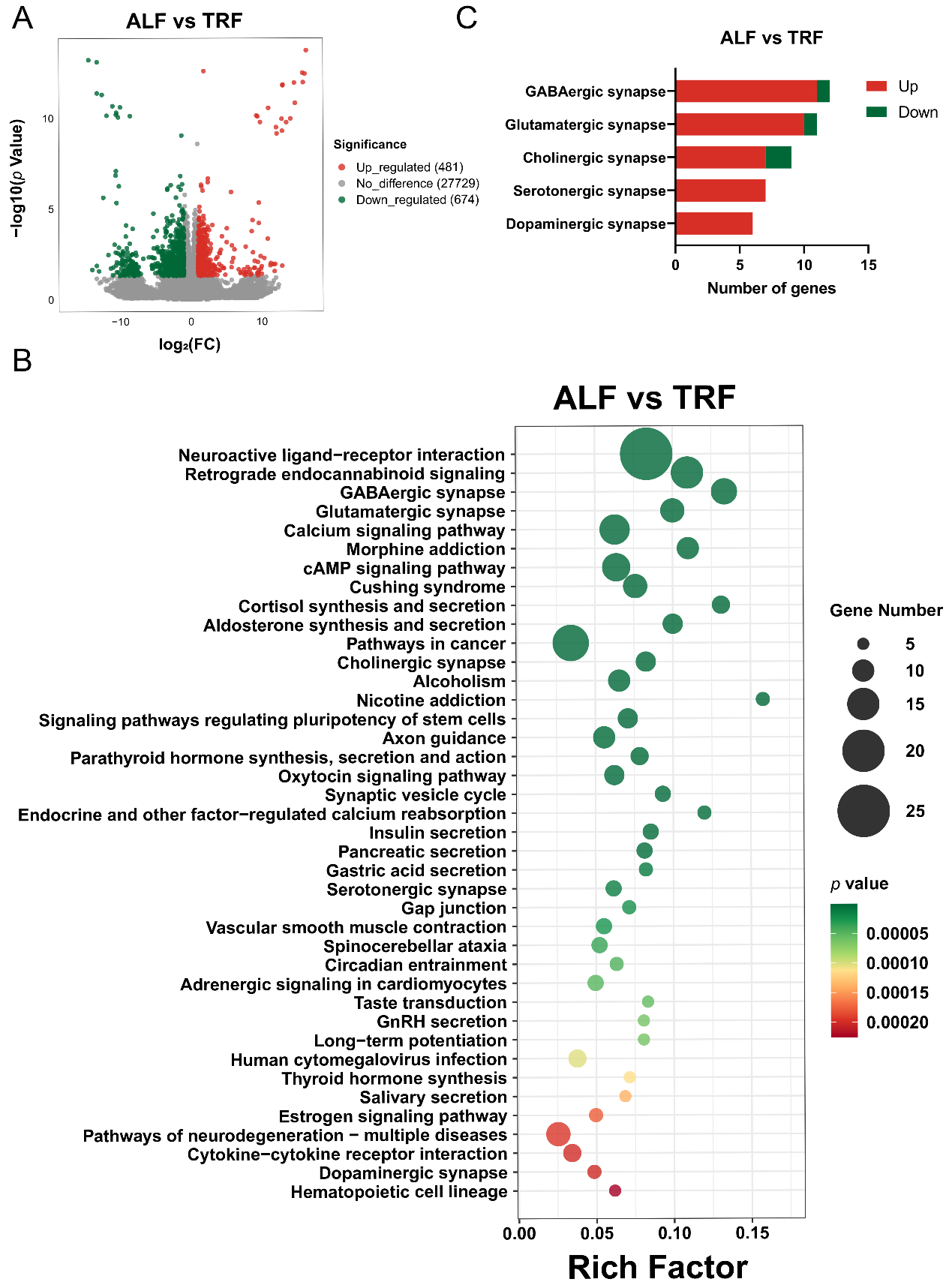


**Figure S2.**


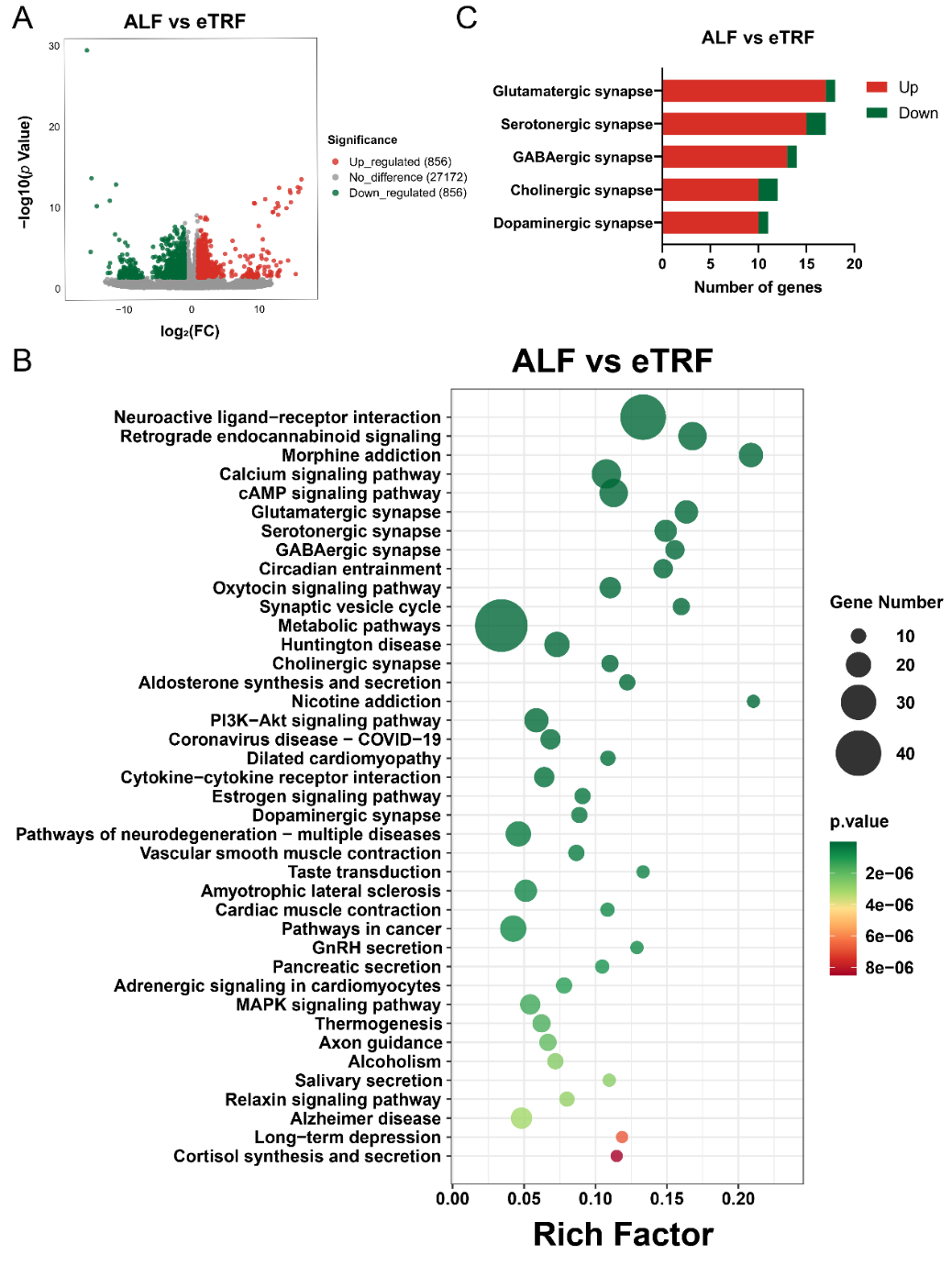


**Figure S3.**


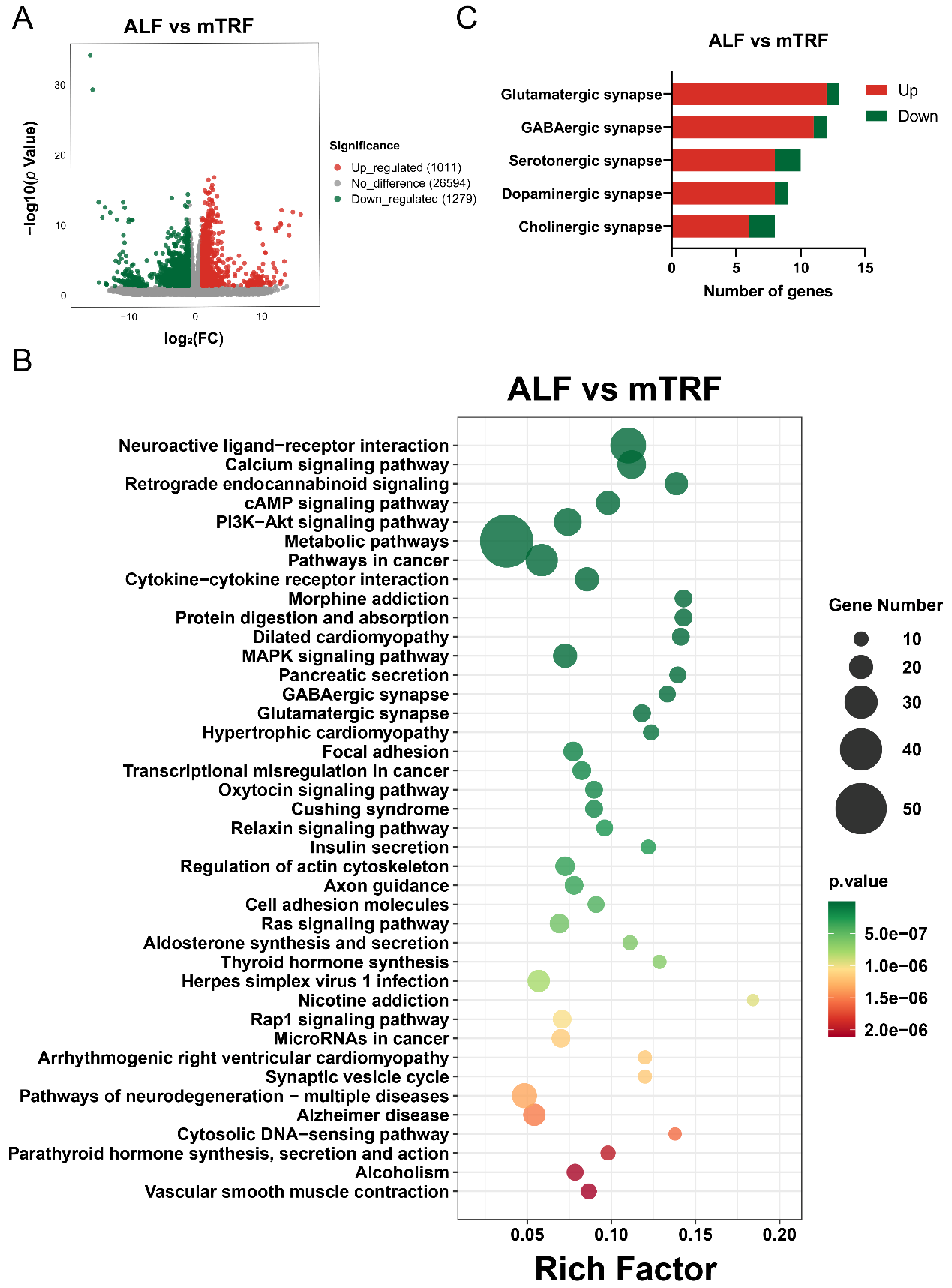


**Figure S4.**


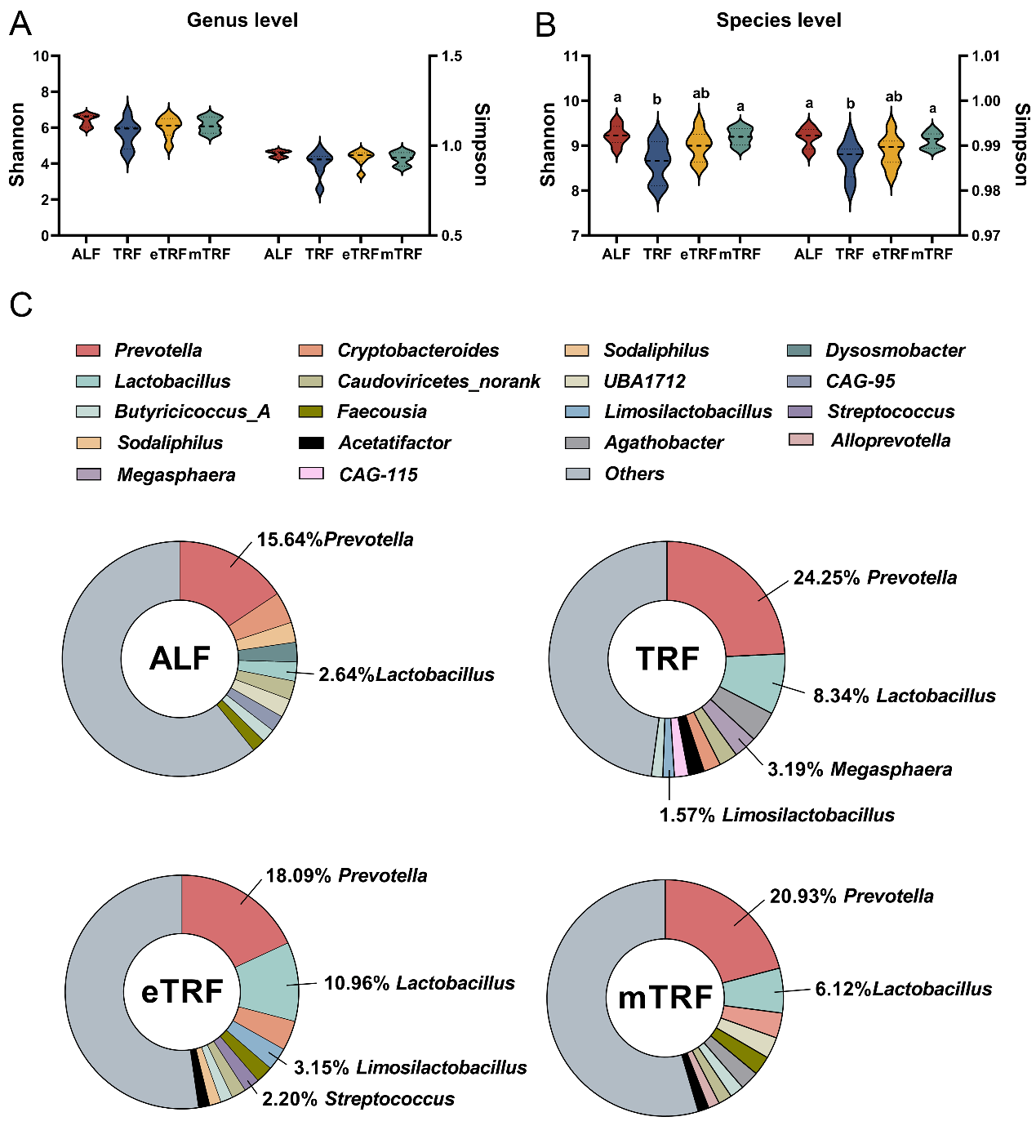

Supplement: Supplemental Material [file KGMI_A_2467185_SM3370.zip › Supplement/Supplement figures.docx]
